# Molecular Epidemiology of Geographically Dispersed *Vibrio cholerae*, Kenya, January 2009–May 2010

**DOI:** 10.3201/eid1806.111774

**Published:** 2012-06

**Authors:** Ahmed Abade Mohamed, Joseph Oundo, Samuel M. Kariuki, Hamadi I. Boga, Shanaz K. Sharif, Willis Akhwale, Jared Omolo, Anyangu S. Amwayi, David Mutonga, David Kareko, Mercy Njeru, Shan Li, Robert F. Breiman, O. Colin Stine

**Affiliations:** Field Epidemiology and Laboratory Training Program, Nairobi, Kenya (A.A. Mohamed, J. Oundo, J. Omolo, A.S. Amwayi);; Ministry of Public Health and Sanitation, Nairobi (A.A. Mohamed, S.K. Sharif, W. Akhwale, J. Omolo, A.S. Amwayi, D. Mutonga, M. Njeru);; Centers for Disease Control and Prevention, Nairobi (J. Oundo, R.F. Breiman);; Kenya Medical Research Institute, Nairobi (J. Oundo, S.M. Kariuki);; Jomo Kenyatta University of Agriculture and Technology, Nairobi (H.I. Boga, D. Kareko);; University of Maryland School of Medicine, Baltimore, Maryland, USA (S. Li, O.C. Stine)

**Keywords:** phenotypes, genotypes, Vibrio cholerae, cholera, characterization, molecular epidemiology, outbreaks, bacteria, Kenya

## Abstract

Numerous outbreaks of cholera have occurred in Kenya since 1971. To more fully understand the epidemiology of cholera in Kenya, we analyzed the genetic relationships among 170 *Vibrio cholerae* O1 isolates at 5 loci containing variable tandem repeats. The isolates were collected during January 2009–May 2010 from various geographic areas throughout the country. The isolates grouped genetically into 5 clonal complexes, each comprising a series of genotypes that differed by an allelic change at a single locus. No obvious correlation between the geographic locations of the isolates and their genotypes was observed. Nevertheless, geographic differentiation of the clonal complexes occurred. Our analyses showed that multiple genetic lineages of *V. cholerae* were simultaneously infecting persons in Kenya. This finding is consistent with the simultaneous emergence of multiple distinct genetic lineages of *V. cholerae* from endemic environmental reservoirs rather than recent introduction and spread by travelers.

## Abstract 2

Isolates represent multiple genetic lineages, a finding consistent with multiple emergences from endemic reservoirs.

Cholera, caused by the bacterium *Vibrio cholerae* and characterized by a profuse watery diarrhea, has been a serious public health problem since the first recorded pandemic in 1817. In 2009, the World Health Organization (WHO) reported just over 220,000 cholera cases and nearly 5,000 cholera-related deaths in 45 countries; however, these estimates are thought to be substantially underestimated because many countries where cholera is endemic do not report cases. During the past 20 years, the highest reported incidence shifted from the Americas to Africa. Africa accounted for 98% of reported cholera cases and 99% of reported cholera-related deaths during 2009.

Kenya has had numerous outbreaks of cholera since the first case was detected there in 1971 (*1*); 15 discrete outbreaks of cholera were documented during 1971–2010 (*2**–**6*). In western Kenya during January–April 2008, a cholera outbreak affected 10 administrative districts in Nyanza Province (adjacent to Lake Victoria), resulting in 790 cases and 53 deaths (case-fatality rate 6.7%) (*6*). The peak of the outbreak (January 2008) occurred after the December 2007 presidential election in Kenya, which had disputed results that triggered periods of protest, violence, public transportation disruption, and work stoppages throughout the country.

After an outbreak subsides in the lake region of Kenya, it has been suggested that *V. cholerae* becomes extinct in that locale but that isolated pockets of disease linger elsewhere in the region, and when the climate becomes favorable, *V. cholerae* reemerges and spreads from the refuge (*7*). The most recent outbreak occurred during January 2009–May 2010; cholera was detected in at least 52 districts throughout the country, and a total of 11,769 cases and 274 deaths (case-fatality rate ≈2.3%) were reported to the Kenya Ministry of Public Health and Sanitation. The regularity of these outbreaks indicates that *V. cholerae* might be frequently spread by travelers or that it is endemic to the area.

Published epidemiologic studies used the best methods available at the time to differentiate isolates of *V. cholerae* in Kenya. For example, several investigations (*8*) used pulsed-field gel electrophoresis (PFGE) to characterize the genetic relatedness of the isolates responsible for cholera outbreaks. These studies found that all of the isolates were closely related, as would be expected if there was a spread from a single source. However, PFGE (*9**,**10*) discriminates poorly among serotype O1 and O139 *V. cholerae* strains. Multilocus-variable tandem repeat analysis (MLVA), however, has been reported to be useful in differentiating *V. cholerae* O1 strains in various rural communities and within households (*11**–**14*). To more fully understand the epidemiology of cholera in Kenya, we used MLVA to characterize the genetic relatedness of *V. cholerae* strains isolated from persons throughout the country.

## Materials and Methods

### Study Regions

**Figure 1:**
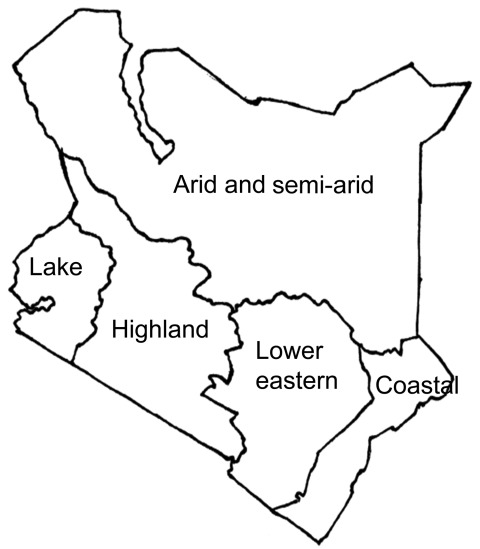
Geographic/climatic regions as defined in a study of the genetic relatedness of O1 *Vibrio cholerae* isolates, Kenya, January 2009–May 2010.

We identified all districts in Kenya that reported cases of cholera during January 2009–May 2010. The Division of Disease Surveillance and Response, Ministry of Public Health and Sanitation, Kenya, provided a list of cases that met a clinical case definition for cholera. For the purpose of this study, we defined cholera as sudden onset of >3 episodes of watery diarrhea in a 24-h period in a person >2 years of age (compared with 5 years of age in the WHO definition) or <2 years of age if a clinician suspected cholera and *V. cholerae* was isolated from a stool specimen (corresponds with the WHO confirmed case definition, http://www.who.int/cholera/technical/prevention/control/en/index1.html). We used records from the provincial headquarters of the affected provinces and districts to update the list. After compiling a complete and up-to-date national list, we divided the country into 5 geographic regions, according to local climate conditions: the coastal region, the arid and semi-arid region, the lake region, the lower eastern region, and the highland region (Figure 1). We estimated the population of these regions by adding the population of districts within each region (data provided by the National Bureau of Statistics, Kenya 2009 Population and Housing Census Highlights, www.knbs.or.ke/Census%20Results/KNBS%20Brochure.pdf).

### Isolation of *V. cholerae* Strains in Kenya

Stool or rectal swab specimens were obtained from 222 persons with suspected cases of cholera who met the clinical case definition during the 2009–2010 cholera outbreak. The specimens were transferred onto Cary-Blair medium and transported (at 2°–8°C) to the Kenya National Public Health Laboratory Services, Nairobi, within 48 hours after collection. They were then cultured (37°C, 8 h) in alkaline peptone water and subcultured (37°C, 18–24 h) on thiosulfate–citrate–bile salts–sucrose agar (HiMedia Laboratories Ltd., Mumbai, India). After the specimens were subcultured, putative *V. cholerae* isolates were examined for sucrose fermentation. Suspicious colonies were subcultured (37°C, 18–24 h) again on Mueller-Hinton agar (Scharlau Chemie, Barcelona, Spain). All isolates were tested for the presence of oxidase, and they were serotyped with a polyvalent O1 antiserum and with monospecific Inaba and Ogawa antisera (Denka Seiken, Tokyo, Japan). All confirmed *V. cholerae* isolates were stored at −80°C in trypticase soy broth (Scharlau Chemie) supplemented with 20% (vol/vol) glycerol.

### Genotyping

Of the 222 stool specimens collected, 173 (78%) yielded *V. cholera* isolates; they were stored at −80°C until use. Of these 173 frozen isolates, 170 were revived by streaking onto Luria-Bertani agar and grown overnight at 37°C. Single, well-isolated colonies were selected to be grown in Luria-Bertani broth overnight at 37°C. We isolated *V. cholerae* DNA from the broth cultures by using PrepMan Ultra (ABI, Foster City, CA, USA) according to the manufacturer’s instructions. We used PCR and primers as described (*13*) to amplify 5 loci containing variable length tandem repeats. Agarose gel electrophoresis was used to confirm the presence of amplified products.

The fluorescent-labeled products were separated and detected by using a model 3730xl Automatic Sequencer (ABI); internal lane standards (Liz600; ABI) and the GeneScan program (ABI) were used to determine produce sizes. Genotypes were determined by using published formulas to calculate the number of repeats from the length of each allele and to order the alleles at the 5 loci. The 5 loci, in order, are VC0147, VC0436–7 (intergenic), VC1650, VCA0171, and VCA0283; thus, the genotype 9,4,6,19,11 indicates that the isolate has alleles of 9, 4, 6, 19, and 11 repeats at the 5 loci, respectively. These standard loci will be in the global *V. cholerae* MLVA database, which is currently being developed (contact jimmyloh@dso.org.sg). Relatedness of the strains was assessed by using eBURSTv3 (http://eburst.mlst.net). Genetically related genotypes were defined as those possessing identical alleles at 4 of the 5 loci. The mismatch amplification mutation PCR was used to screen for the cholera toxin–encoding gene, as described (*15*).

## Results

### Epidemiology

During January 2009–May 2010, 11,769 cases of cholera were reported to the Division of Disease Surveillance and Response, Kenyan Ministry of Public Health and Sanitation. Demographic and geographic information was obtained for 10,497 of the case-patients, of whom 246 (2.3%) died and 173 (1.65%) had laboratory-confirmed cholera. Case-patients who met the clinical case definition were reported from 52 (35%) of the country’s 149 districts and from all 5 geographic regions (Table 1). The age range for case-patients was 1–76 years (overall mean 23 years, SD ± 18 years). The attack rate ranged from 0.02% in the lake and highland regions to 10-fold higher (0.2%) in the arid and semi-arid region. Case fatality rates ranged from 0.7% in the coastal region to 4.0% in the arid and semi-arid region (Table 1).

**Table 1:** Cholera attack rate and CFR by geographic/climatic region, Kenya, January 2009–May 2010*

Region	No. cases	Mean age ± SD, y (range)	Attack rate, %†	No. deaths (CFR, %)
Coastal	1,484	21 ± 18 (1–70)	0.07	10 (0.7)
Highland	1,139	28 ± 18 (1–75)	0.02	46 (4.0)
Arid and semi-arid	4,210	25 ± 19 (1–70)	0.20	146 (3.5)
Lake	1,019	25 ± 17 (1–76)	0.02	23 (2.3)
Lower eastern	2,645	25 ± 18 (1–67)	0.12	21 (2.3)
Total	10,497	23 ± 18 (1–76)	0.07	246 (2.3)

*CFR, case-fatality rate. †Denominator was based on population as provided by the National Bureau of Statistics (Kenya 2009 Population and Housing Census Highlights, www.knbs.or.ke/Census%20Results/KNBS%20Brochure.pdf).

Cases of cholera were reported almost every day during January 2009–May 2010, and the number of cases reported during any 1 day ranged from 1 to 160 for the entire country. The first reported case was from the lake region (reported on January 2, 2009), and index cases appeared during the next 2 months in each of the other 4 regions. In the highland (including Nairobi), arid and semi-arid North, lower eastern, and coastal regions, the index cases were reported on February 22, February 26, March 12, and May 28, respectively. Every index case heralded the start of an apparent unified outbreak in the respective regions (Technical Appendix), during which time, the number of cases progressively increased. For example, in the lake, arid and semi-arid, highland, and lower eastern regions, the first outbreak peak occurred on January 20, April 7, May 5, and November 4, respectively. After the initial flush of cases in each region, the number of cases per week decreased to few or none; however, additional peaks, with numerous cases per week, would occur later.

### Genetic Relatedness

#### Classical ctxB, Biotype and Serotype

Consistent with the possible spread of *V. cholerae* across the country, each of the 170 isolates was biotype El Tor and carried the classical *ctxB* allele, as measured by mismatch amplification mutation PCR. In contrast, 84% of the isolates were serotype Inaba, and the other 16% were serotype Ogawa.

#### Multilocus-Variable Tandem Repeat Analysis

MLVA of the *V*. *cholerae* O1 isolates revealed extensive genetic diversity. To determine whether the strains were genetically related, we genotyped all 170 isolates at 5 loci containing variable numbers of tandem repeats. All loci exhibited substantial variation; for example, VC0147, VC436–7 (intergenic), VC1650, VCA0171, and VCA0283 had 9, 4, 6, 21, and 24 alleles, respectively. When each isolate was assigned a genotype (on the basis of and in order of the number of repeat units at each locus), 106 genotypes were detected among the 170 isolates. eBURSTv3 analysis to determine the genetic relatedness of the genotypes revealed 5 clonal complexes, each comprising a series of genotypes that differed by an allelic change at a single locus. In addition, we detected 19 singleton genotypes that were unrelated to any other genotype; that is, they differed at >2 loci from all other genotypes. The arid and semi-arid region yielded 42% (8 isolates) of these singletons; the coast, highland, and lake regions contributed 26% (5 isolates), 21% (4 isolates), and 11% (2 isolates), respectively. No singletons were detected in the lower eastern region.

**Figure 2:**
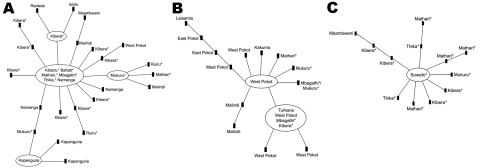
Genetic relatedness of O1 *Vibrio cholerae* isolates from an outbreak of cholera, Kenya, January 2009–May 2010. The 3 largest clonal complexes are shown; smaller clonal complexes consisted of 2 or 3 genotypes. A) Clonal complex 1 was observed in each geographic/climatic region. B) Clonal complex 2 was detected in the Rift Valley (western part of the arid and semi-arid region) and in the coastal, lower eastern, and highland (primarily in informal settlements around Nairobi, represented by asterisks) regions. C) Clonal complex 3 was detected in the coastal, lake, and highland regions and in the Rift Valley (arid and semi-arid region).Genetic relatedness was determined by using eBURSTv3 (http://eburst.mlst.net/). Each genotype is represented by a node in the diagram; each connecting line represents an allelic change at a single locus.

The 3 largest clonal complexes (designated 1, 2, and 3) occurred throughout most of the country. Clonal complex 1 contained 52 different genotypes among 89 isolates (Figure 2, panel A), and it was observed in every region (Table 2). The most common genotype, identified as the founder genotype (defined as the genotype that differed from the largest number of other genotypes at only 1 locus), was observed in 22 isolates collected from informal settlements around Nairobi. The founder genotype radiated into 12 other genotypes, and 7 of those differentiated further. No distinct correlation between the geographic locations and genotype of the isolates was detected.

**Table 2:** Number of *Vibrio cholerae* isolates, by clonal complex and geographic/climatic region, Kenya, January 2009–May 2010

Region	Clonal complex, no. isolates					
	1	2	3	4	5	Singletons
Arid and semi-arid	17	20	4	0	2	8
Highland	50	8	17	2	0	4
Lower eastern	7	5	0	0	0	0
Coastal	10	2	1	0	0	7
Lake	1	0	1	0	0	2
Total	85	35	23	2	2	21

Clonal complex 2, which contained 20 different genotypes among 33 isolates (Figure 2, panel B), was detected in the Rift Valley (in the western part of the arid and semi-arid region) and in the coastal, lower eastern, and highland (primarily represented by informal settlements around Nairobi) regions. Clonal complex 3, which contained 11 genotypes among 23 isolates (Figure 2, panel C) was detected in the coastal, lake, and highland regions and in the Rift Valley (arid and semi-arid region).

Some geographic differentiation of the clonal complexes appears to have occurred (Table 2). The 3 large clonal complexes were distributed in a statistically significant (p<0.0002, by 3 × 4 χ^2^ test), nonrandom manner across the arid and semi-arid, coastal, highland, and lower eastern regions (the lake region had too few isolates to be tested). This significant difference can be attributed to the finding that clonal complex 2 was the most common complex in the arid and semi-arid region, and clonal complexes 1 and 3 were the most common complexes in the highland region. These findings are consistent with our observation that clonal complexes 4 and 5 were each seen in only 1 region, but they are very small groups. Clonal complex 4 isolates were found during various months; however, the complex 5 isolates were found only during February 2009, which was during the beginning of the outbreak in Moyale, an isolated village on the border with Ethiopia.

**Figure 3:**
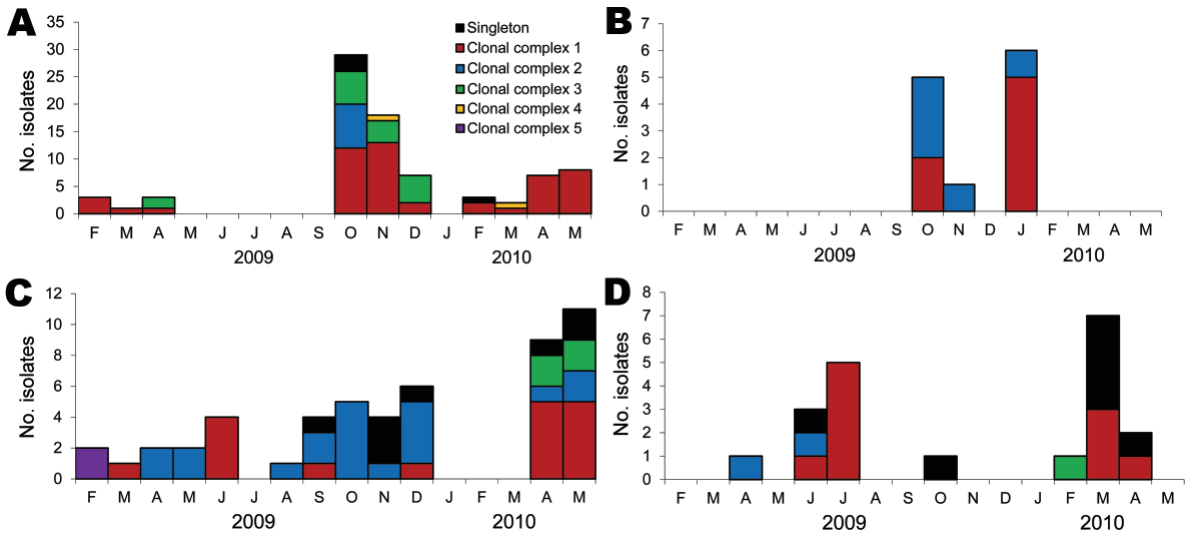
Distribution of *Vibrio cholerae* isolates, by clonal complex and month of isolation, Kenya, January 2009–May 2010. A) Highland region, including informal settlements around Nairobi. B) Coastal region. C) Arid and semi-arid region. D) Lower eastern region.

Figure 3 shows the temporal distribution of the 5 genetically distinct clonal complexes and the singletons for 4 regions; no temporal separation of the various clonal complexes is apparent. Isolates collected in February 2009 from the highland region and arid part of the arid and semi-arid region belong to distinct clonal complexes, 1 and 5, respectively; however, isolates collected in March 2009 from the same 2 regions all belong to clonal complex 1. Substantial variation was observed early in the outbreak: 2 or 3 clonal complexes are represented in the first 7 isolates genotyped from each region. In the highlands region, every 3-month period in which isolates were found has >2 distinct genotypes (Figure 3, panel A). In the coastal region, every month >1 isolate assayed has >2 genotypes (Figure 3, panel B). In the arid and semi-arid region, April and May 2009, when 4 isolates were assayed, are the only 2 consecutive months with a single genotype (Figure 3, panel C). Even in the lower eastern region, where only 13 isolates were assayed, multiple genotypes were observed in the 2 months in which >1 isolate was found (Figure 3, panel D).

## Discussion

The results of our genetic analyses suggest that the most recent outbreak of cholera in Kenya (beginning in January 2009) represented several distinct genetic lineages of *V. cholerae* that emerged simultaneously, perhaps facilitated by environmental and behavioral factors around the country. Thus, these data suggest that these outbreaks likely resulted from endemic foci rather than from recent introduction and spread by travelers.

In contrast, previous outbreaks of cholera in Kenya have been attributed to the spread of *V. cholerae* by travelers. Outbreaks in the 1990s and in 2005 were attributed to a clone carried by travelers to Nairobi; from there, it spread to other locations in the country (*4*). Likewise, the well-publicized 2010 cholera outbreak in Haiti also appears to have resulted from the introduction of *V. cholerae* by travelers (*16*). In addition, there have been repeated introductions of *V. cholerae* into previously disease-free locations by travelers from Southeast Asia and other areas where cholera is endemic (*17*). On a smaller scale, *V. cholerae* is thought to persist around the African Great Lakes Region because, although it becomes extinct in areas, it is still present in other localities and can spread when weather conditions are favorable (*7*).

Genetic uniformity is expected when *V. cholerae* is spread from a single source. This expectation conflicts with the observation that isolates with Inaba and Ogawa serotypes occurred during the outbreak. However, the expectation is consistent with the genetic evidence that all isolates had the El Tor biotype and identical (by Tenover’s criteria) (*18*) PFGE patterns (J.O. Oundo, et al. unpub. data). The nearly identical (single band) differences in the PFGE patterns are consistent with whole genome sequencing results showing that the toxigenic El Tor lineage of O1 *V. cholerae* has few large (several kilobytes) insertions or deletions (*19*) and few nucleotide changes (*17*). Large insertions or deletions or nucleotide changes in restrictions sites are the mutations that produce altered PFGE patterns; their absence in the El Tor lineage is consistent with the minimal number of PFGE patterns.

All *V. cholerae* strains that we studied from the 2009–2010 outbreak in Kenya had the classical *ctx*B allele, which is consistent with a clonal origin. The earlier population of *V. cholerae* is presumed to have contained only the El Tor allele because the classical *ctx*B allele was not detected before 1989 in isolates with the El Tor background (*20*). Thus, the presence of the classical allele in all isolates from the 2009–2010 outbreak most likely represents a rapid shift in allele frequency. Such a shift occurred in O1 *V. cholerae* in Kolkata, India, when allele frequency changed from 100% El Tor in 1989 to 100% classical *ctx*B in 1995 (*20*). It is of critical clinical importance that, the classical *ctx*B allele, when found in the background of an El Tor strain, has been associated with a more severe form of cholera than that caused by a strain with the El Tor *ctx*B allele (*21**,**22*).

Our MLVA genotyping results for 170 *V. cholerae* isolates from the 2009–2010 outbreak in Kenya showed extensive genetic diversity: we found 5 clonal complexes and 19 singleton genotypes. In contrast, studies using PFGE found genetic uniformity among *V. cholerae* isolates from 1994 to 2010 (*5**,**8*) (J.O. Oundo, et al. unpub. data). These differences may be explained by the finding that MLVA is superior to PFGE for discriminating between isolates of *V. cholerae* O1 (*11**–**13*).

An alternative explanation for the substantial variation observed among *V. cholerae* isolates from Kenya is that their tandem repeat loci may result from a rapid accumulation of mutations. However, 3 findings have documented the relative stability of those loci. First, an analysis of the variation occurring during a month-long serial passage revealed that the 3 large chromosomal loci (the primary determinants of membership in the various clonal complexes) were largely stable (*13*). Second, the clonal complexes remained distinct during 3 consecutive years in Bangladesh (*13*), longer than the 17 months of the 2009–2010 outbreak in Kenya. Third, all isolates from the cholera outbreak that began in October 2010 in Haiti belonged to a single clonal complex (*16*). These observations and the co-occurrence of distinct clonal complexes are consistent with the idea that there were multiple simultaneous outbreaks of cholera in Kenya.

Diverse MLVA genotyping results are found in countries where *V. cholerae* is endemic. In India, 6 clonal complexes were detected in *V. cholerae* O1 isolates (*23*), and in Bangladesh, 7 clonal complexes were detected (*12*). Consistent with these findings, we detected 5 clonal complexes in isolates from Kenya. The genetic diversity is also consistent with data from single-nucleotide polymorphism analyses that showed the several distinct waves of immigration of *V. cholerae* into Kenya (*17*), if the descendants of these immigrants settled and survived. The presence of multiple distinct lineages across the country supports the notion that there have been multiple introductions of *V. cholerae* and that over time these strains spread countrywide. The multiplicity of clonal complexes is in stark contrast to the apparent uniformity of the *ctx*B allele. An earlier study demonstrated that the *ctxB* alleles, as part of a mobile genetic element, were found in distinct locations in different MLVA clonal complexes (*14*).

The timing of the observed diversity is consistent with multiple separate outbreaks and not with the spread of disease by travelers. In February 2009, isolates from highland region, including Nairobi, belonged to clonal complex 1 and might have spread from the lake region. However, isolates from Moyale in the arid and semi-arid region belonged to clonal complex 5, and the time between the initial observations in the southern part of the country and the far north was too short for the isolates to have evolved into a new clonal complex. In addition, the far north is a region where travel is difficult and people from south seldom travel. Thus, these 2 outbreaks appear to be temporally, geographically, and genetically separate. As stated, the mutations necessary for 1 clonal complex to evolve into another are not expected to occur in a few months. Thus, during the 2009–2010 outbreak, the occurrence of isolates from a second complex in the highland region (clonal complex 3) and the arid and semi-arid region (clonal complex 2) cannot be explained by evolutionary changes. In addition, no source was found from which travelers could have introduced these *V. cholerae* strains. A similar argument can be made for clonal complex 4 isolates and for each of the singletons scattered across the country. That is, the time to their appearance was too short to represent an evolutionary change, and there was no source from which a traveler spread the genetically distinct isolates. Thus, our analyses led to the conclusion that *V. cholerae* is endemic in Kenya. The extensive genetic variation among the isolates exposes a limitation in our sampling scheme. Our sampling should have included many more isolates from each region and each time period. The observed genetic differences permit distinguishing between regions, but the small number of samples limits what can be inferred about what is happening within a region.

Kenya has experienced multiple major outbreaks of cholera, and different regions of the country have reported different attack rates. During the 2009–2010 outbreak, all of the isolates collected contained the classical *ctxB* allele. Despite this genetic uniformity, our MLVA results showed that the isolates had extensive genetic variation within and between geographic locations. The genetic relatedness studies we performed showed that 5 clonal complexes and 106 different genotypes were part of the outbreak; thus, *V. cholerae* had several genetic lineages. Our data are consistent with *V. cholerae* isolates being endemic throughout Kenya.

## Supplementary Material

Technical Appendix Time curve of cholera outbreak in Kenya, January 2009–August 2010.

## References

[1] Scrascia M, Maimone F, Mohamud KA, Materu SF, Grimont F, Grimont PA, Clonal relationship among *Vibrio cholerae* O1 El Tor strains causing the largest cholera epidemic in Kenya in the late 1990s. J Clin Microbiol. 2006;44:3401–4. 10.1128/JCM.00611-06 16954285 PMC1594678

[2] Tauxe RV, Mintz ED, Quick RE. Epidemic cholera in the new world: translating field epidemiology into new prevention strategies. Emerg Infect Dis. 1995;1:141–6. 10.3201/eid0104.950408 8903186 PMC2626892

[3] Shapiro RL, Otieno MR, Adcock PM, Phillips-Howard PA, Hawley WA, Kumar L, Transmission of epidemic *Vibrio cholerae* O1 in rural western Kenya associated with drinking water from Lake Victoria: an environmental reservoir for cholera? Am J Trop Med Hyg. 1999;60:271–6. 10072150 10.4269/ajtmh.1999.60.271

[4] Scrascia M, Pugliese N, Maimone F, Mohamud KA, Ali IA, Grimont PA, Cholera in Ethiopia in the 1990 s: epidemiologic patterns, clonal analysis, and antimicrobial resistance. Int J Med Microbiol. 2009;299:367–72. 10.1016/j.ijmm.2008.10.004 19121605

[5] Mugoya I, Kariuki S, Galgalo T, Njuguna C, Omollo J, Njoroge J, Rapid spread of *Vibrio cholerae* O1 throughout Kenya, 2005. Am J Trop Med Hyg. 2008;78:527–33. 18337355

[6] Shikanga OT, Mutonga D, Abade M, Amwayi S, Ope M, Limo H, High mortality in a cholera outbreak in western Kenya after post-election violence in 2008. Am J Trop Med Hyg. 2009;81:1085–90. 10.4269/ajtmh.2009.09-0400 19996441

[7] Nkoko DB, Giraudoux P, Plisnier PD, Tinda AM, Piarroux M, Sudre B, Dynamics of cholera outbreaks in great lakes region of Africa, 1978–2008. Emerg Infect Dis. 2011;17:2026–34. 10.3201/eid1711.110170 22099090 PMC3310557

[8] Kiiru JN, Saidi SM, Goddeeris BM, Wamae NC, Butaye P, Kariuki SM. Molecular characterisation of *Vibrio cholerae* O1 strains carrying an SXT/R391-like element from cholera outbreaks in Kenya: 1994–2007. BMC Microbiol. 2009;9:275. 10.1186/1471-2180-9-275 20040104 PMC2806261

[9] Kurazono H, Yamasaki S, Ratchtrachenchai O, Nair GB, Takeda Y. Analysis of *Vibrio cholerae* O139 Bengal isolated from different geographical areas using macrorestriction DNA analysis. Microbiol Immunol. 1996;40:303–5. 8709866 10.1111/j.1348-0421.1996.tb03350.x

[10] Basu A, Garg P, Datta S, Chakraborty S, Bhattacharya T, Khan A, *Vibrio cholerae* O139 in Calcutta, 1992–1998: incidence, antibiograms, and genotypes. Emerg Infect Dis. 2000;6:139–47. 10.3201/eid0602.000206 10756147 PMC2640858

[11] Danin-Poleg Y, Cohen LA, Gancz H, Broza YY, Goldshmidt H, Malul E, *Vibrio cholerae* strain typing and phylogeny study based on simple sequence repeats. J Clin Microbiol. 2007;45:736–46. 10.1128/JCM.01895-06 17182751 PMC1829105

[12] Stine OC, Alam M, Tang L, Nair GB, Siddique AK, Faruque SM, Seasonal cholera from multiple small outbreaks, rural Bangladesh. Emerg Infect Dis. 2008;14:831–3. 10.3201/eid1405.071116 18439375 PMC2600222

[13] Kendall EA, Chowdhury F, Begum Y, Khan AI, Li S, Thierer JH, Relatedness of *Vibrio cholerae* O1/O139 isolates from patients and their household contacts, determined by multilocus variable-number tandem-repeat analysis. J Bacteriol. 2010;192:4367–76. 10.1128/JB.00698-10 20585059 PMC2937383

[14] Choi SY, Lee JH, Jeon YS, Lee HR, Kim EJ, Ansaruzzaman M, Multilocus variable-number tandem repeat analysis of *Vibrio cholerae* O1 El Tor strains harbouring classical toxin B. J Med Microbiol. 2010;59:763–9. 10.1099/jmm.0.017939-0 20299504

[15] Morita M, Ohnishi M, Arakawa E, Bhuiyan NA, Nusrin S, Alam M, Development and validation of a mismatch amplification mutation PCR assay to monitor the dissemination of an emerging variant of *Vibrio cholerae* O1 biotype El Tor. Microbiol Immunol. 2008;52:314–7. 10.1111/j.1348-0421.2008.00041.x 18577166

[16] Ali A, Chen Y, Johnson JA, Redden E, Mayette Y, Rashid MH, Recent clonal origin of cholera in Haiti. Emerg Infect Dis. 2011;17:699–701. 21470464 10.3201/eid1704.101973 PMC3377427

[17] Mutreja A, Kim DW, Thomson NR, Connor TR, Lee JH, Kariuki S, Evidence for several waves of global transmission in the seventh cholera pandemic. Nature. 2011;477:462–5. 10.1038/nature10392 21866102 PMC3736323

[18] Tenover FC, Arbeit RD, Goering RV. How to select and interpret molecular strain typing methods for epidemiological studies of bacterial infections: a review for healthcare epidemiologists. Molecular Typing Working Group of the Society for Healthcare Epidemiology of America. Infect Control Hosp Epidemiol. 1997;18:426–39. 10.1086/647644 9181401

[19] Chun J, Grim CJ, Hasan NA, Lee JH, Choi SY, Haley BJ, Comparative genomics reveals mechanism for short-term and long-term clonal transitions in pandemic Vibrio cholerae. Proc Natl Acad Sci U S A. 2009;106:15442–7. 10.1073/pnas.0907787106 19720995 PMC2741270

[20] Raychoudhuri A, Patra T, Ghosh K, Ramamurthy T, Nandy RK, Takeda Y, Classical ctxB in *Vibrio cholerae* O1, Kolkata, India. Emerg Infect Dis. 2009;15:131–2. 10.3201/eid1501.080543 19116078 PMC2660696

[21] Siddique AK, Nair GB, Alam M, Sack DA, Huq A, Nizam A, El Tor cholera with severe disease: a new threat to Asia and beyond. Epidemiol Infect. 2010;138:347–52. 10.1017/S0950268809990550 19678971

[22] Nair GB, Qadri F, Holmgren J, Svennerholm AM, Safa A, Bhuiyan NA, Cholera due to altered El Tor strains of *Vibrio cholerae* O1 in Bangladesh. J Clin Microbiol. 2006;44:4211–3. 10.1128/JCM.01304-06 16957040 PMC1698305

[23] Ghosh R, Nair GB, Tang L, Morris JG, Sharma NC, Ballal M, Epidemiological study of *Vibrio cholerae* using variable number of tandem repeats. FEMS Microbiol Lett. 2008;288:196–201. 10.1111/j.1574-6968.2008.01352.x 18811655

