# Supplementary material for: Molecular Epidemiology of Geographically Dispersed Vibrio cholerae, Kenya, January 2009–May 2010
Source: Emerg Infect Dis. 2012 Jun;18(6):925–31. doi: 10.3201/eid1806.111774 (PMC3358164; doi:10.3201/eid1806.111774)
Supplement: Technical Appendix — Time curve of cholera outbreak in Kenya, January 2009–August 2010. [file 11-1774-Techapp_1p.pdf]

Article DOI: http://dx.doi.org/10.3201/eid1806.111774

Molecular Epidemiology of Geographically Dispersed *Vibrio cholerae*, Kenya, January 2009–May 2010

Technical Appendix

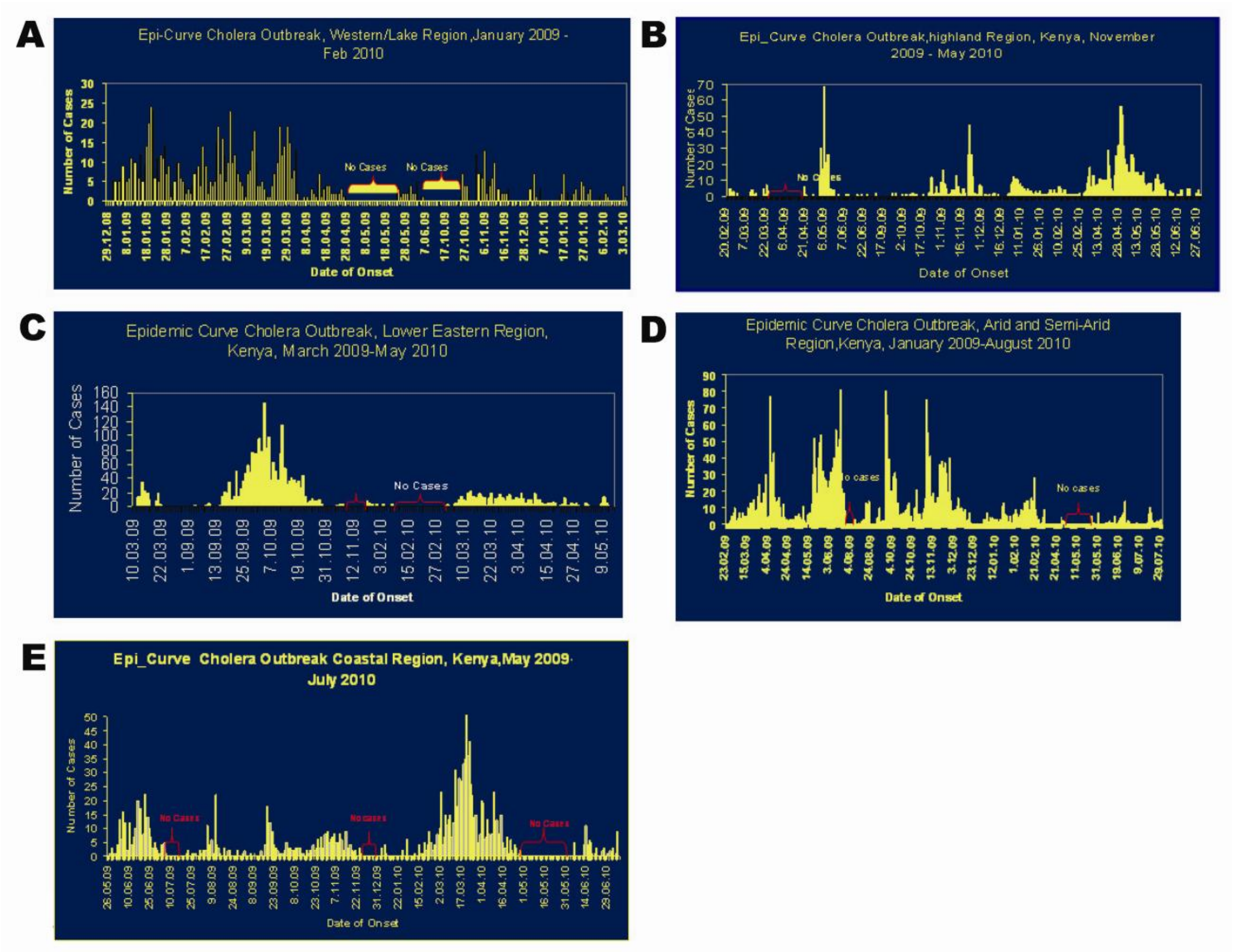

Technical Appendix Figure. Time curve of cholera outbreak in Kenya, January 2009–August 2010. A) Lake region, January 2009–February 2010. B) Highland region, including informal settlements around Nairobi, November 2009–May 2010. C) Lower eastern region, March 2009–May 2010. D) Arid and semi-arid region, January 2009–August 2010. E) Coastal region, May 2009–July 2010.
